# Inflammatory and cardiac-stress biomarkers in Moyamoya disease: the role of GDF-15

**DOI:** 10.3389/fnagi.2026.1906016

**Published:** 2026-10-12

**Authors:** Fengxia Guo, Hanxiao Li, Qiushi Guo, Yumeng Wang, Xiaohuan Zhang, Weiguang Luo

**Affiliations:** Department of Clinical Laboratory, Henan Provincial People’s Hospital, People’s Hospital of Zhengzhou University, Zhengzhou, Henan, China

**Keywords:** cardiac-stress, GDF-15, inflammatory biomarker, Moyamoya disease, neutrophils

## Abstract

**Background:**

Moyamoya disease (MMD) is an uncommon cerebrovascular condition that requires the identification of easily measurable biomarkers to enable prompt diagnosis. This study aimed to: (1) compare clinical and laboratory parameters between individuals with MMD and control subjects; (2) examine the associations of these parameters with growth differentiation factor-15 (GDF-15); (3) evaluate the diagnostic effectiveness of potential biomarkers; and (4) identify independent risk factors for MMD.

**Methods:**

The study cohort comprised 265 participants, including 121 patients diagnosed with MMD and 144 healthy control subjects. The parameters measured included white blood cell count (WBC), neutrophil count (NEU), Glu, homocysteine (HCY), GDF-15, and soluble growth stimulation expressed gene 2 (ST-2) concentrations. The analyses conducted encompassed correlation assessments, receiver operating characteristic (ROC) curve evaluations, and multivariable logistic regression analyses.

**Results:**

Baseline characteristics were comparable between groups except for smoking status, which was significantly more frequent in the MMD group (*p* = 0.046). Patients diagnosed with MMD exhibited significantly elevated levels of WBC, NEU, HCY, GDF-15 and soluble ST-2. No significant differences were observed in Glu levels (*p* = 0.468). GDF-15 demonstrated a positive correlation with WBC (*r* = 0.53, *p* < 0.0001), NEU (*r* = 0.45, *p* = 0.026), but not with Glu or HCY (*p* > 0.05). ROC analysis revealed that GDF-15 had the highest area under the curve (AUC) (0.779), followed by NEU (0.668), Glu (0.590), and WBC (0.577). Multivariable logistic regression analysis identified Diabetes, NEU, and GDF-15 as independent risk factors (all *p* < 0.001). A 100 pg./mL increment in GDF-15 levels was correlated with an estimated 49% elevation in the odds of MMD occurrence.

**Conclusion:**

Patients diagnosed with MMD exhibit a unique profile of inflammatory and cardiac-stress biomarkers. GDF-15 and NEU were independently correlated with MMD. Among these, GDF-15 demonstrated the highest diagnostic efficacy, suggesting its potential utility as a non-invasive adjunctive biomarker.

## Introduction

MMD is a rare, progressive cerebrovascular disorder characterized by idiopathic stenosis or occlusion of the terminal segment of the internal carotid arteries. This condition results in the development of a compensatory, fragile collateral vascular network at the base of the brain, which is visualized as a “puff of smoke” on angiographic imaging (Mertens et al., 2025). The disease is associated with an increased risk of recurrent ischemic or hemorrhagic strokes, leading to significant neurological deficits and a reduced quality of life (Haas et al., 2024; Zhang et al., 2025; Berry et al., 2020). Despite advancements in surgical revascularization techniques, early diagnosis remains challenging due to the often-non-specific nature of initial symptoms (Mertens et al., 2025; Graf et al., 2019). The diagnostic methodology for MMD can be divided into three complementary modalities. Firstly, invasive angiographic diagnosis through digital subtraction angiography (DSA) remains essential for the identification of unilateral lesions and the differentiation of MMD from atherosclerotic disease. Secondly, noninvasive structural diagnosis, facilitated by characteristic MRI and MRA findings, is now permissible under the 2021 revised diagnostic criteria in appropriate cases (Kuroda et al., 2022). Thirdly, functional hemodynamic assessment can be conducted using noninvasive MRI-based techniques, such as oxygen-dependent MRI, which have been employed to evaluate cerebrovascular reactivity in MMD patients (He et al., 2024). Despite these advancements, neuroimaging is limited by factors such as cost, availability, and the need for specialized expertise, making it impractical for large-scale screening, early risk stratification, or serial monitoring of disease progression. Therefore, the development of non-invasive and easily measurable peripheral blood biomarkers for early screening, risk stratification, and monitoring of disease progression remains a critical clinical priority (Li et al., 2026; Guo et al., 2025).

Recent research underscores the pivotal role of inflammation in the pathogenesis of MMD. Numerous studies have reported elevated levels of inflammatory cytokines, chemokines, and markers of endothelial activation in patients with MMD compared to healthy controls (Xia et al., 2025; Yasuda et al., 2026). Furthermore, recent cellular and vascular investigations, including single-cell analyses of affected MMD vessels, have provided direct evidence of immune-vascular interactions, endothelial alterations, phenotypic changes in vascular smooth muscle cells, and pathological vascular remodeling in MMD (He et al., 2025a). These findings imply that both systemic inflammatory responses and localized vascular inflammatory mechanisms are integral to the pathogenesis of MMD.

Among the potential biomarkers, GDF-15 has garnered considerable interest. GDF-15 is a stress-responsive cytokine within the transforming growth factor-β superfamily, and its expression is upregulated in response to cellular stress, inflammation, oxidative stress, and hypoxia (Afzal et al., 2026; Wischhusen et al., 2020). Elevated levels of GDF-15 have been observed in various cardiovascular diseases, where it is regarded as a marker of adverse outcomes (Sawalha et al., 2023; Tektonidou et al., 2021; Sonner et al., 2026). Nevertheless, the precise role of GDF-15 in MMD has yet to be comprehensively elucidated. Furthermore, systemic immune-inflammatory markers, including the neutrophil-to-lymphocyte ratio (NLR), platelet-to-lymphocyte ratio (PLR), lymphocyte-to-monocyte ratio (LMR), and systemic inflammatory index (SII), have been linked to the prognosis of various cardiovascular diseases. Considering the association of MMD with inflammatory processes (Guo et al., 2023; Liu et al., 2023), the relationship between GDF-15 and these inflammatory markers in the context of MMD remains insufficiently understood.

Consequently, the current study was formulated with the following objectives: (1) to compare conventional laboratory parameters, including WBC, NEU, glucose, and HCY, as well as GDF-15 and ST-2, between patients with MMD and matched control subjects; (2) to investigate the correlations between GDF-15 and other inflammatory and metabolic markers; (3) to assess the diagnostic efficacy of potential biomarkers in differentiating MMD from controls through ROC curve analysis; and (4) to identify independent risk factors for MMD using multivariable logistic regression analysis.

## Methods

### Study population

This retrospective case–control study systematically enrolled 121 patients diagnosed with MMD and 144 healthy control subjects between June 2025 and December 2025. The eligibility criteria for patient inclusion were as follows: (1) participants had to be 18 years of age or older; (2) a confirmed diagnosis of MMD was required, established through digital subtraction angiography (DSA) or high-resolution magnetic resonance angiography (HR-MRI/MRA), in accordance with the diagnostic criteria outlined in the 2024 Edition of the Chinese Expert Consensus on the Diagnosis and Treatment of Moyamoya Disease and Moyamoya Syndrome; and (3) the diagnosis had to have been made within the preceding 6 months, with no prior history of symptomatic cerebrovascular events, such as transient ischemic attacks, cerebral infarction, or cerebral hemorrhage. The exclusion criteria were delineated as follows: (1) the presence of conditions with established etiologies that could precipitate secondary Moyamoya syndrome, including but not limited to atherosclerotic stenosis (ascertained through comprehensive vascular wall imaging), autoimmune disorders (such as systemic lupus erythematosus and antiphospholipid syndrome), meningitis, a history of cranial radiotherapy, neurofibromatosis type I, and Down syndrome; (2) a history of cranial or extracranial vascular reconstructive surgery; (3) the presence of concurrent severe hepatic or renal impairment (characterized by ALT/AST levels exceeding three times the upper limit of normal, or an eGFR<30 mL/min/1.73m^2^), active malignancy, acute and chronic infections, uncontrolled thyroid dysfunction, or other conditions significantly impacting metabolic or inflammatory status; (4) long-term use (defined as a duration of 1 month or more) of lipid-lowering medications (such as statins, fibrates, or PCSK9 inhibitors), or the receipt of systemic immunosuppressive therapy prior to enrollment; and (5) pregnancy or lactation. All serum samples used in this study were residual specimens obtained from routine clinical laboratory testing. MMD patient samples were collected at the time of diagnosis. Control samples were obtained from age- and sex-matched healthy individuals who underwent routine health check-ups during the same period. No additional blood draws or procedures were performed specifically for this study. Blood samples were obtained at the point of diagnosis, preceding the commencement of any anticoagulant or antiplatelet therapy, to mitigate potential confounding effects of these medications on inflammatory biomarker profiles. This study was approved by the Ethics Committee of Henan Provincial People’s Hospital (Approval No. 2025(108)). All serum samples used in this study were residual specimens obtained from routine clinical testing. Written informed consent was obtained from all participants prior to their inclusion in the study. All methods were performed in accordance with the relevant guidelines and regulations.

### Clinical parameters and laboratory tests

The fundamental clinical data of the patients, encompassing sex, age, pertinent medical history (including hypertension, diabetes, smoking, and alcohol consumption), along with other baseline characteristics, were systematically collected. Participants underwent a fasting period of 10 h prior to blood collection. Following complete coagulation, the samples were centrifuged at 3000 revolutions per minute for 10 min, and the supernatant was subsequently extracted for analysis. The concentrations of alanine aminotransferase (ALT), aspartate aminotransferase (AST), creatinine (CREA), uric acid (UA), urea (UREA), glucose (Glu), and HCY were quantified utilizing an Abbott Fully Automated C1600 analyzer. WBC and NEU counts were assessed using a Sysmex XN-10 Automated Hematology Analyzer (Sysmex Corporation, Kobe, Japan). Serum levels of GDF-15 and ST-2 were measured with an i3000 automatic chemiluminescence analyzer (Maccura Biotechnology Co., Ltd., Chengdu, China), employing the reagents and quality control materials provided by the manufacturer.

### Statistical analysis

Statistical analyses were performed using SPSS version 26.0. The normality of continuous variables was evaluated using the Shapiro–Wilk test. Normally distributed variables were presented as mean ± standard error of the mean (SEM), and variables that did not follow a normal distribution were expressed as median (interquartile range) and analyzed using the Mann–Whitney U test. Unpaired Student’s *t*-test was used to assess differences between the two groups for normally distributed variables. Categorical variables were described in terms of frequencies (percentages) and compared using either the chi-square test or Fisher’s exact test, depending on the suitability. Correlation analyses between GDF-15 and continuous clinical parameters were conducted utilizing Pearson’s correlation coefficient for variables exhibiting a normal distribution and Spearman’s rank correlation coefficient for variables that do not follow a normal distribution. ROC curve analysis was employed to evaluate the discriminatory efficacy of potential biomarkers in distinguishing patients with MMD from control subjects, with the AUC and 95% confidence intervals (CI) being calculated. Multivariable logistic regression analysis was performed to identify independent risk factors associated with MMD. A two-sided *p*-value of less than 0.05 was considered to indicate statistical significance for all tests. All *p*-values were calculated directly from the raw individual data.

## Results

### Baseline clinical and laboratory parameters of enrolled subjects

As illustrated in Table 1, the baseline characteristics of the two groups were predominantly similar. There were no statistically significant differences observed in variables such as sex, Age, Hypertensive, Alcohol, Diabetes, ALT, AST, CREA, UA, or UREA. Nonetheless, the proportion of smokers was notably higher in the MMD group compared to the control group.

**Table 1:** Baseline clinical and laboratory parameters in enrolled subjects.

Characteristics	Control (*n* = 144)	MMD (*n* = 121)	*p* value
Male/Female	65/79	48/73	0.385
Age (years)	46 ± 3.29	45 ± 2.98	0.276
Smoking [*n* (%)]	54 (37%)	61 (50%)	0.046
Hypertensive [*n* (%)]	61 (42%)	50 (41%)	0.900
Alcohol [*n* (%)]	38 (26%)	29 (24%)	0.672
Diabetes [*n* (%)]	26 (18%)	30 (24%)	0.226
ALT (U/L)	18.32 ± 2.79	19.16 ± 1.67	0.343
AST (U/L)	18.21 ± 3.40	17.91 ± 1.15	0.290
CREA (umol/L)	57.83 ± 6.57	59.09 ± 4.33	0.561
UA (umol/L)	324.71 ± 12.61	334.28 ± 20.3	0.330
UREA (mmol/L)	6.14 ± 1.09	6.24 ± 1.12	0.394

Data are mean ± standard error of the mean (SEM).

### Biomarker levels in controls and patients with MMD

As demonstrated in Table 2, individuals diagnosed with MMD showed significantly increased levels of WBC, NEU, and HCY compared to the control group (all *p* < 0.05). In contrast, Glu levels did not differ significantly between groups (*P*>0.05). Additionally, the levels of GDF-15 and ST-2 were markedly elevated in the MMD group. GDF-15 concentrations did not differ between non-smokers and smokers within the MMD group (*P*>0.05, Table 3).

**Table 2:** Biomarker levels in controls and patients with MMD.

Characteristics	Control (*n* = 144)	MMD (*n* = 121)	*p* value
WBC (10^9^/L)	5.49 ± 0.19	6.47 ± 0.32	<0.0001
NEU (10^9^/L)	3.26 ± 0.18	4.06 ± 0.05	<0.0001
Glu (mmol/L)	5.23 ± 0.09	5.35 ± 0.14	0.468
HCY (umol/L)	10.61 ± 0.38	12.40 ± 0.83	0.032
GDF-15 (pg/mL)	371.22 ± 19.16	701.31 ± 37.42	<0.0001
ST-2 (ng/mL)	7.55 ± 1.35	11.16 ± 1.14	0.0063

Data are mean ± standard error of the mean (SEM).

**Table 3:** Comparison of serum GDF-15 levels between non-smokers and smokers in the MMD group.

Characteristics	Non-smoking (*n* = 60)	Smoking (*n* = 61)	*p* value
GDF-15 (pg/mL)	710.5 ± 68.46	702.2 ± 44.86	0.917

### Correlations between GDF-15 and inflammatory/metabolic parameters

A correlation analysis was conducted to investigate the associations between GDF-15 and clinical biomarkers. As illustrated in Table 4, GDF-15 exhibited a positive correlation with WBC and NEU. Conversely, no statistically significant correlations were identified between GDF-15 and Glu or HCY.

**Table 4:** Correlation analysis between GDF-15 and clinical parameters.

GDF-15	WBC	NEU	Glu	HCY
*r*	0.53	0.45	0.08	0.04
*P*	0.0001	0.026	0.389	0.578

Analyses were performed in the MMD group (*n* = 121) using Pearson’s correlation coefficient. *r*, correlation coefficient.

### ROC analysis of GDF-15, WBC, NEU, and Glu for distinguishing MMD from controls

The diagnostic efficacy of potential biomarkers for distinguishing patients with MMD from control subjects was evaluated through ROC curve analysis. GDF-15 demonstrated the most significant discriminatory power, as evidenced by AUC of 0.779 (95% CI: 0.723–0.834; *p* < 0.0001). At the optimal threshold of 405 pg./mL, the sensitivity and specificity were 75.21 and 75.00%, respectively. NEU also demonstrated significant diagnostic utility, achieving an AUC of 0.668 (95% CI: 0.599–0.728, *p* < 0.0001). This was followed by Glu, which presented an AUC of 0.590 (95% CI: 0.5217–0.6601, *p* = 0.0106), and WBC, with an AUC of 0.577 (95% CI: 0.5096–0.6457, *p* = 0.0287) (Figure 1).

**Figure 1:**
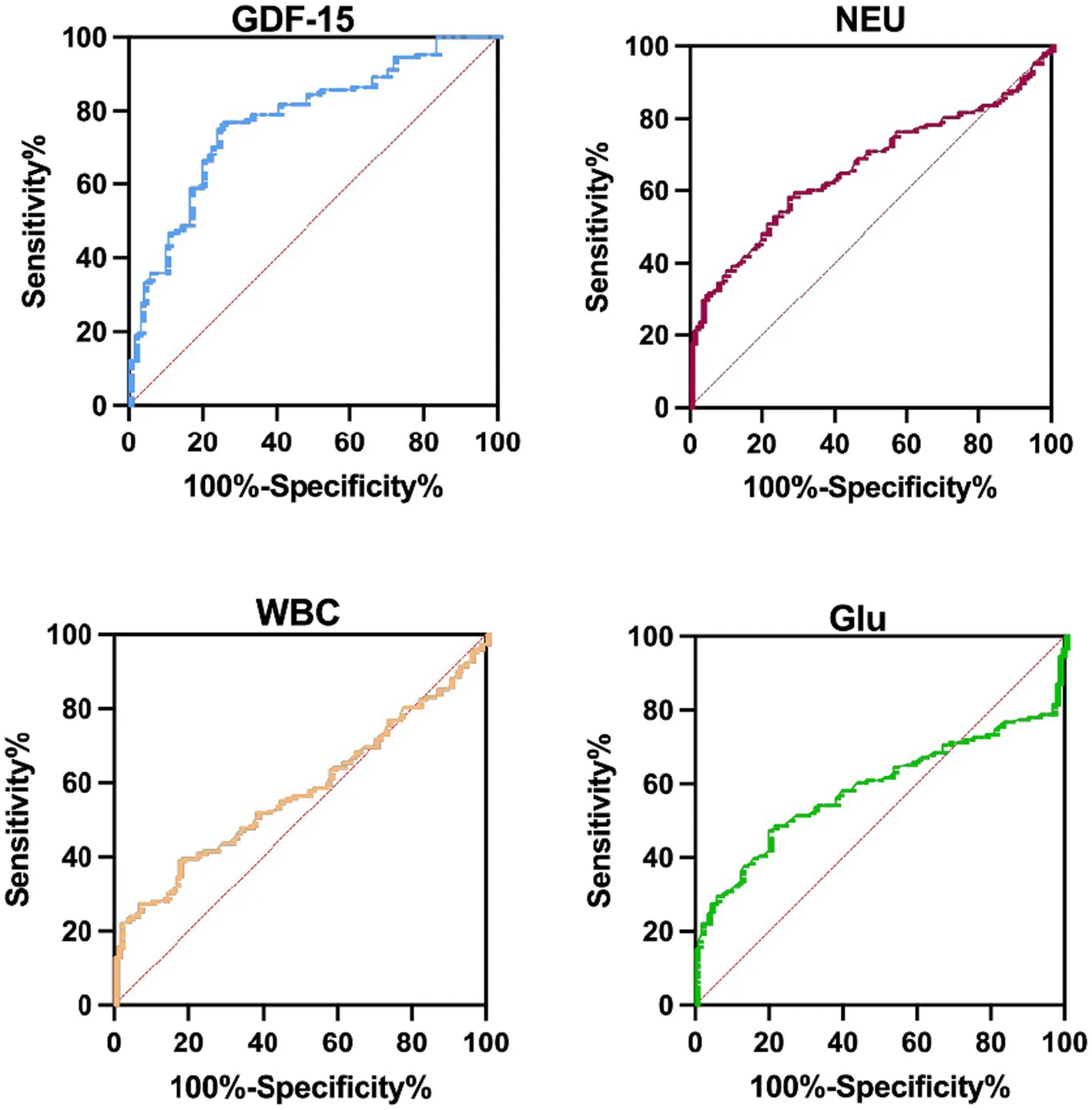
ROC curves evaluating the diagnostic performance of GDF-15, NEU, WBC, and Glu. **(A)** GDF-15 (blue line). **(B)** NEU (purple line). **(C)** WBC (orange line). **(D)** Glu (green line). Sensitivity (%) is plotted against 100%-Specificity (%). The red dashed line indicates the reference line.

### Multivariable logistic regression analysis of factors associated with MMD

As demonstrated in Table 5, Diabetes, NEU and GDF-15 were found to be significantly associated with the presence of MMD. Conversely, Gender, Age, Hypertension and Smoking (*p* = 0.166) did not present as significant predictors.

**Table 5:** Multivariable logistic regression analysis of factors associated with MMD.

Variables	Gender	Age	Hypertension	Diabetes	Smoking	NEU	GDF-15
SE	0.29	0.01	0.29	0.31	0.29	0.13	0.001
OR	0.81	1.004	1.78	2.86	1.51	1.85	1.004
95%CI	0.45–1.4	0.97–1.01	1.002–3.19	1.55–5.42	0.84–2.72	1.45–2.44	1.003–1.006
*P*	0.474	0.635	0.051	0.0009	0.166	<0.0001	<0.0001

## Discussion

In this study, we conducted a systematic comparison of a range of inflammatory, metabolic, and cardiac-stress biomarkers between patients with MMD and healthy control subjects. We assessed the diagnostic performance of these biomarkers and identified independent risk factors associated with MMD. The principal findings of our research are as follows: (1) Patients with MMD exhibited significantly elevated levels of WBC, NEU, HCY, GDF-15, and ST-2 compared to the control group; (2) GDF-15 demonstrated a correlation with WBC, NEU; (3) GDF-15 showed the highest discriminatory capacity with an AUC of 0.779, followed by NEU; and (4) Diabetes, NEU, and GDF-15 were identified as independent risk factors for MMD.

The marked elevation in WBC and NEU counts among patients with MMD lends support to the emerging hypothesis that chronic low-grade inflammation is associated with MMD. Neutrophils, as pivotal components of the innate immune system, have been implicated in endothelial dysfunction, vascular remodeling, and thrombosis in general cardiovascular pathology (Gomez-Moreno et al., 2018; Li et al., 2025). Prior research has documented an increased NLR in MMD (Wu et al., 2025), and our findings build upon previous observations by demonstrating that the NEU count is independently associated with MMD. However, it is important to note that our data indicate a systemic inflammatory association rather than providing direct evidence of neutrophil involvement in the vascular remodeling associated with MMD. The vascular pathology of MMD is characterized by coordinated alterations in endothelial cells, vascular smooth muscle cells (VSMCs), circulating molecular signals, and immune cell interactions. Notably, disease-specific experimental evidence has shown that serum-related mechanisms can induce phenotypic switching of VSMCs in MMD (He et al., 2025b). Furthermore, spatial proteomic analysis of occluded MMD arteries has revealed intimal infiltration of synthetic VSMCs and antigen-presenting immune cells (Asselman et al., 2025). These findings support the hypothesis that circulating inflammatory signals may play a role in vascular remodeling in MMD, although the precise contribution of neutrophils to this process remains to be elucidated.

In our study, GDF-15 emerged as the potential adjunctive biomarker, exhibiting the largest fold-change between groups, the highest area under the ROC curve at 0.779, and a correlation with WBC (*r* = 0.53). Each 100 pg./mL increase in GDF-15 was associated with a ~ 49% increase in the odds of MMD. GDF-15 is typically upregulated in response to oxidative stress, hypoxia, and inflammation across various cardiovascular and ischemic conditions (Wollert et al., 2017). Elevated circulating levels of GDF-15 have also been documented in other cerebrovascular disorders, such as ischemic stroke and cerebral small vessel disease, where they are indicative of adverse prognoses (Wu et al., 2024; Zang et al., 2022; Dong and Nao, 2019). In the context of MMD, specific studies have identified alterations in circulating molecules, pathological angiogenesis, and vascular remodeling (He et al., 2023a,b). However, the direct involvement of GDF-15 in these MMD-specific mechanisms has yet to be elucidated. To the best of our knowledge, this is the first study to demonstrate that GDF-15 levels are significantly elevated in MMD and may serve as a potential adjunctive biomarker. The observed correlation with WBC suggests that GDF-15 partially mirrors the systemic inflammatory burden; however, its superior AUC suggests an additional contribution that is independent of systemic inflammation. Further mechanistic investigations are necessary to elucidate whether this additional contribution reflects neuronal injury, vascular remodeling, or other disease-specific mechanisms in MMD.

ST-2, a constituent of the interleukin-1 receptor family, is a recognized biomarker indicative of cardiac stress and fibrosis (Dong and Nao, 2019; Zhang et al., 2020; Dikme et al., 2021). The significant elevation of ST-2 levels observed in patients with MMD indicates a potential correlation between MMD and subclinical cardiac or systemic vascular stress, a correlation that has not been documented in prior research. Further studies are necessary to elucidate whether ST-2 plays a direct role in the pathophysiology of MMD or if its elevation is merely an epiphenomenon. In our cohort, although ST-2 showed a significant difference in univariate analysis, it was not included in the subsequent ROC analysis or multivariable regression. This decision was informed by the primary objective of assessing GDF-15 in conjunction with traditional inflammatory parameters, as well as by our aim to prevent multicollinearity and overfitting due to the limited occurrence of MMD events. The statistical signal for ST-2 was also relatively weaker than that for GDF-15. Nevertheless, the significant univariate finding for ST-2 is intriguing and warrants independent validation in future prospective studies with larger cohorts and pre-specified designs to evaluate its potential additive or interactive value with GDF-15.

The ROC analysis indicated that GDF-15 exhibits fair to good diagnostic accuracy in differentiating MMD from control subjects, with an area under the ROC curve of 0.779. Establishing a cutoff value that achieves high sensitivity and specificity may prove clinically beneficial for screening populations at elevated risk. NEU demonstrated moderate discriminatory power, with an AUC of 0.668, whereas glucose and WBC showed limited diagnostic utility, with AUCs approximately 0.590 and 0.577, respectively. The relatively low AUCs associated with these conventional markers underscore the necessity for the development of novel potential adjunctive biomarker, such as GDF-15. Although GDF-15 demonstrated the highest AUC value of 0.779 among the markers assessed in this study, it is important to recognize that this value, despite being statistically significant, is inadequate for GDF-15 to function as an independent diagnostic test in clinical settings. Typically, an AUC greater than 0.90 is deemed necessary for a screening tool to be clinically viable. Consequently, our findings should be regarded as preliminary and hypothesis-generating, indicating that GDF-15 warrants further exploration as a potential adjunctive, non-invasive biomarker, rather than as a substitute for neuroimaging or a definitive diagnostic test.

Multivariable logistic regression analysis identified diabetes, NEU, and GDF-15 as independent risk factors for MMD. The association with diabetes is particularly noteworthy. Although MMD is traditionally not classified as a diabetic complication, diabetes may exacerbate microvascular dysfunction and inflammation, potentially accelerating the progression of MMD (Ergul et al., 2014; Umemura et al., 2017). Hypertension demonstrated a borderline association, consistent with previous studies that have identified hypertension as a common comorbidity in MMD (Liu et al., 2022; Lee et al., 2024). Regarding smoking status, in our study, we conducted a comparative analysis of serum GDF-15 levels between smokers and non-smokers within the MMD cohort, revealing no statistically significant difference (*p* = 0.917). This finding further corroborates the notion that smoking does not substantially influence serum GDF-15 levels in our study population. While univariate analysis indicated a higher prevalence of smoking within the MMD group (*p* = 0.046), smoking did not emerge as an independent predictor in the multivariable model (*p* = 0.166). This suggests that the association between smoking and MMD may be mediated through alternative inflammatory or metabolic pathways.

This study is subject to several limitations that merit consideration. Firstly, the sample size was relatively small and sourced from a single center, lacking an independent external validation cohort. Secondly, the cross-sectional case–control design limits the ability to assess whether GDF-15 or other biomarkers are associated with disease severity or long-term progression, and it precludes the drawing of causal inferences; longitudinal studies are necessary to ascertain whether elevated levels of GDF-15 and NEU precede the onset of the disease or result from it. Thirdly, we did not stratify patients with MMD according to clinical subtype (ischemic versus hemorrhagic) or disease stage (Suzuki grade). Additionally, our inclusion criteria, which focused on patients diagnosed within the preceding 6 months and with no history of symptomatic cerebrovascular events, were deliberately applied to minimize confounding effects from acute post-stroke inflammatory responses. Nevertheless, this implies that our cohort is representative of a particular subset of MMD patients, specifically those with newly diagnosed, asymptomatic conditions. Considering that a significant number of MMD patients present with ischemic or hemorrhagic stroke, our findings may not be directly applicable to the entire MMD population. Consequently, our results should be regarded as exploratory and hypothesis-generating. Future large-scale, multicenter prospective studies involving larger cohorts, including patients with diverse clinical presentations and stages of the disease, are necessary to validate and expand upon our observations.

## Data Availability

The raw data supporting the conclusions of this article will be made available by the authors, without undue reservation.
